# How One-Shot Learning Unfolds in the Brain

**DOI:** 10.1371/journal.pbio.1002138

**Published:** 2015-04-28

**Authors:** Janelle Weaver

**Affiliations:** Freelance Science Writer, Carbondale, Colorado, United States of America

## Abstract

Much learning occurs gradually through trial and error, but rare and important experiences require one-shot learning; a new study explores how the brain switches between these two strategies for identifying causal relationships. Read the Research Article.

Imagine you go out to a restaurant and order oysters for the first time, then for the main course you order your usual plate of chicken. Later that evening, your stomach becomes upset and you start to develop hives on your skin. Because you have eaten chicken many times before without becoming ill, you conclude after this one experience that the oysters caused your symptoms.

When humans and other animals encounter an outcome they have never previously experienced, it may be necessary to rapidly learn the link between cause and effect in order to survive. After experiencing two events that are paired together for the first time, animals can quickly learn that one caused the other. This phenomenon, called one-shot learning, is very different from incremental learning, in which new knowledge is acquired gradually through trial and error. While much evidence supports the notion of dissociable memory systems for one-shot and incremental learning, little is known about how one-shot learning unfolds at the neural level or how the brain is capable of switching between different types of learning strategies.

In a study published in this issue of *PLOS Biology*, Sang Wan Lee, John O’Doherty, and Shinsuke Shimojo of the California Institute of Technology combined computational, behavioral, and brain-imaging techniques to shed light on these questions. According to the authors, their model is the only framework to date that is optimized to account for one-shot learning. Their evidence suggests that the hippocampus is selectively switched on when one-shot learning is predicted to occur, and the ventrolateral prefrontal cortex may act as a switch to turn on and off one-shot learning as required (Fig 1).

**Fig 1:**
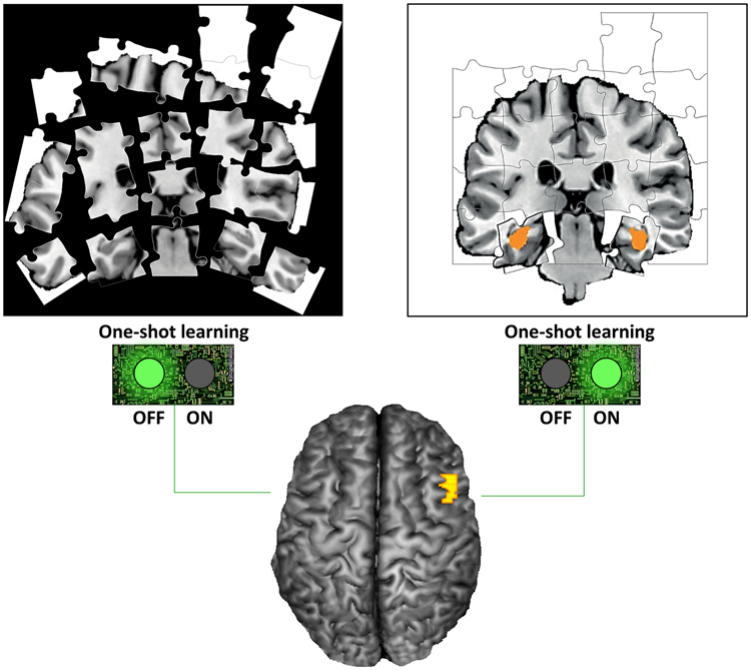
Ventrolateral prefrontal cortex mediates rapid causal inference. Here, the authors use a combination of computational modeling and neuroimaging techniques to provide evidence indicating that the ventrolateral prefrontal cortex, in co-operation with the hippocampus, underlies switching between incremental and one shot learning. *Image credit*: *Sang Wan Lee*.

Their novel computational framework suggests that individuals learn causal relationships more quickly when they are less certain about whether one event caused another. The authors reasoned that when the uncertainty about cause and effect is high, more focus is required to learn the causal relationship, resulting in the high learning rate associated with one-shot learning. To test their computational hypothesis, the researchers analyzed behavioral data and functional magnetic resonance imaging (fMRI) data acquired from individuals who performed a simple causal inference task in which learning could occur from a single experience. In each trial, participants were presented with a sequence of pictures followed by a positive or negative number reflecting a monetary outcome. At the end of each round of trials, participants were then asked to rate the likelihood that individual pictures would cause particular outcomes; these ratings were used to determine causal uncertainty. If a participant learned the association between a picture and an outcome that were paired together only one time, that trial was considered a one-shot learning event.

When the researchers tested whether their causal uncertainty model best explains the behavioral data, they found that it outperformed all seven alternative learning models typically used to account for incremental learning. Analysis of the fMRI data revealed that higher causal uncertainty was associated with increased activation of the ventrolateral prefrontal cortex, while the hippocampus was activated during one-shot learning events but not incremental learning events. Moreover, activity in the ventrolateral prefrontal cortex was coupled to hippocampal activity for very high learning rates but not learning rates associated with more incremental learning. This finding suggests that the ventrolateral prefrontal cortex uses knowledge about causal uncertainty to control a switch that turns on the hippocampus when one-shot learning is needed.

Taken together, the findings suggest that the brain may solve the problem of one-shot learning by computing uncertainty about the causal relationship between events and adjusting learning rates to accommodate rapid learning about those events. Developing a detailed account of when rapid learning takes place and which brain areas are engaged in this process might shed new light on more efficient learning strategies, in addition to superstitions, delusional reasoning, and erroneous attributions of an outcome to the wrong cause. The study may ultimately have substantial implications for real-world situations such as medical diagnoses, lawsuit cases, and psychiatric diseases, such as schizophrenia.
